# Modelling the potential impact of food taxes based on nutrient and energy content in the UK: a simulation study

**DOI:** 10.1017/S000711452400182X

**Published:** 2025-03-28

**Authors:** Danying Li, Mathilde Gressier, Zoe Hill, Jack Olney, Victoria Targett, Michelle Young, Franco Sassi

**Affiliations:** 1 Section for Nutrition Research, Department of Metabolism, Digestion and Reproduction, Faculty of Medicine, Imperial College London, London, UK; 2 Centre for Health Economics & Policy Innovation, Department of Economics & Public Policy, Imperial College Business School, Imperial College London, London, UK; 3 Office for Health Improvement & Disparities, Department of Health and Social Care, London, SW1H 0EU, UK

**Keywords:** Fiscal policy, Food taxes, Sugar reduction, Modelling

## Abstract

Building on the success of the Soft Drinks Industry Levy (SDIL), new tax proposals have been considered in the public health policy debate in the UK. To inform such debate, estimates of the potential impacts of alternative tax scenarios are of critical importance. Using a modelling approach, we studied the effects of two tax scenarios: (1) a hypothetical excise tax designed to tax food products included in the Sugar Reduction Programme (SRP), accounting for pack size to reduce the convenience of purchasing larger quantities at once; (2) an ad valorem tax targeting products based on the UK Nutrient Profile Model (NPM). Simulations of scenario 1 show a reduction in sugar purchased of up to 38 %, with the largest decreases observed for sweet confectionery with a tiered tax, similar in structure to the SDIL. Expected food reformulation in scenario 1 led to further decreases in sugar purchased for all categories. In scenario 2, under the assumption that the tax would not affect purchases of healthier products, a 20 % tax on less healthy products would reduce total sugar purchased by 4·3 % to 14·7 % and total energy by 4·7 % to 14·8 %. Despite some limitations and assumptions, our results suggest that new fiscal policy options hold a significant potential for improving diet quality beyond what has been achieved by the SDIL and SRP. An estimated increase in consumer expenditures in both scenarios suggests that attention needs to be paid to potentially regressive effects in the design of any new food taxes.

Over the past decade, the prevalence of overweight, obesity and non-communicable diseases (NCD), such as type 2 diabetes, heart disease and some cancers, have reached alarming levels in the UK. According to the Health Survey for England 2021, 26 % of adults in England have obesity (defined as a BMI of 30 or higher), and a further 38 % are overweight (BMI between 25 and 30). Morbidity and mortality associated with obesity and NCD represent a large share of the burden of disease, especially in the post-COVID-19 pandemic era. Unhealthy diets and physical inactivity, raised blood pressure, blood sugar and cholesterol, together with smoking and harmful use of alcohol, are underlying risk factors for common NCD. In turn, NCD have profound effects on individuals’ welfare, including a significant economic burden due to decreased productivity and high health and social care expenditure. Early interventions and population-wide approaches, such as health taxes that encourage changes in consumption behaviours closely associated with NCD risk, can prevent or delay NCD.

Health taxes are levied on products that can harm the health of those who consume them (e.g. tobacco and alcohol products, sugar-sweetened beverages, or foods high in fat, sugar or salt) and aim at providing incentives for consumers to reduce their consumption and for manufacturers to change the products they put on the market.

In the UK, several non-fiscal initiatives have been adopted to improve the population’s diet quality before considering fiscal policies, including the use of a Nutrient Profile Model (NPM) to limit the range of foods that can be advertised during children’s TV programming and front-of-pack labelling. Another initiative was the establishment by the Food Standards Agency (FSA), in 2006, of a salt reduction programme that aimed to reduce the average population salt intake. This programme relied on the achievement of voluntary reformulation targets for key categories of processed foods by all sectors of the food industry. Following this success, Public Health England (PHE) implemented a Sugar Reduction Programme (SRP) in 2016/17, which set a voluntary reformulation ambitions for the food industry – a 20 % reduction by 2020 – and established a monitoring framework for food product categories that typically contribute to high dietary sugar intakes^(1)^.

The Soft Drinks Industry Levy (SDIL) was implemented in the UK in 2018 as a measure to reduce the consumption of sugar from sugar-sweetened beverages^(2)^. The SDIL is levied on manufacturers and importers of beverages with added sugar, that contain 5 g or more total sugar per 100 ml, with the aim of incentivising the beverage industry to reformulate their products to reduce sugar content or offer low-sugar alternatives. A UK study found that, between 2015 and 2020, the total volume of sugar sold per capita per day from soft drinks has declined by 46 %, and more recent data show that between 2015 and 2020, sales of soft drinks in the top sugar tier of SDIL fell by 57·4 %, sales of drinks in the mid-sugar tier by 81·6 % and sales of drinks below the SDIL threshold increased by 65·7 %^(3)^ Research shows that most of the reduction in sugar sales was due to reformulation^(4)^. From 2015 to 2020, the overall sales of soft drink increased whilst the sugar content decreased, providing evidence that improvement in public health could be in line with successful business practices. Data from the National Diet and Nutrition Survey shows that sugar intakes have fallen for some age groups, and that this appears to be driven by soft drinks contributing less to intakes, likely as a result of the changes made to drinks included in the SDIL^(5)^.

This study was designed to estimate, using a modelling approach, the potential benefits of two innovative fiscal policy interventions and to investigate the impact of health taxes on the amounts of sugar and other nutrients purchased in Great Britain (GB). The study relies on a large dataset of food and drink purchases that covers about 30 000 British households. The study also investigates, through modelling, the potential of innovative fiscal policies in helping consumers to reduce purchases of less healthy food and ultimately improving population health. Possible modelling scenarios were considered with PHE. The choice of interventions was based on PHE’s priorities as well as the following considerations: for scenario 1, taxing food based on sugar content aiming at reducing consumer’s purchase of sugar and incentivising product reformulation, leveraging the successful precedent set by the SDIL; for scenario 2, we looked beyond taxing sugar content and, instead, focused on the overall nutrient profiling score of foods. This innovative intervention takes into account multiple aspects of a food’s nutritional value. It has the potential of encouraging consumers to purchase healthier products and manufacturers to improve the overall nutritional quality of their products. The current simulation study examines two promising and innovative fiscal policy interventions, and it does not attempt to model all potential fiscal policy interventions.

## Methods

The scenarios in this study investigate innovative solutions that could potentially address the issues associated with taxing food based on its sugar content, like the SDIL does with beverages, or based on its nutrient profiling score. In scenario 1, a hypothetical excise tax was designed to tax food products containing sugar in GB that are included in the SRP according to pack size. An exercise tax is a tax that is levied on specific goods, and it is often applied to items considered harmful or non-essential. It is a fiscal tool that could be used to promote public health. It is typically calculated as a fixed amount per unit or a specific rate per quantity. In this scenario, smaller packs of products are taxed at a lower rate than larger packs of the same food, aiming to incentivise the switch to smaller packs as they would be cheaper. In contrast, scenario 2 takes a broader perspective and simulates the effect of an ad valorem tax targeting ‘less healthy products’ based on their overall nutrient content (energy, saturated fat, sugar, Na, fruit, vegetable and nut content, fibre and protein) instead of only the sugar content, using the UK NPM^(6)^. An ad valorem tax is a tax that is based on the value of an item and, therefore, is applied to the price of less healthy products in scenario 2 as a percentage. In both scenarios, changes in demand and subsequently in the purchase of food categories that were subject to a hypothetical tax were determined based on price elasticities. Due to data limitations, price elasticities were sourced from a previous study^(7)^.

### Dataset

This study uses data from Kantar Worldpanel, an international consumer research company that collects and analyses data from a panel of 30 000 British households that record all items purchased and brought into the home. The panel is representative of the GB population in terms of household size, number of children, socio-economic status, age group and geographic location. Data are collected through participants recording food products purchased and brought home, who also supply receipts at 4-week intervals. Kantar Worldpanel collects the quantities and expenditure of foods purchased by households and adds information on the nutrient composition of each of the foods purchased based on food labels. It is worth noting that the data do not take into account any food waste, and all food purchased and consumed out of homes. This study covers food categories included in the SRP, namely biscuits, breakfast cereals, cakes, chocolate confectionery, ice cream, morning goods, puddings, sweet spreads and sauces, sweet confectionery and yogurts. The dataset includes data on information of each food product, such as pack size and nutrient composition, as well as estimated household annual purchases of each food product, aggregated at country level, including total expenditures, total sugar and energy purchased. Kantar Worldpanel 2019 data were used in the study. The STROBE-nut reporting guidelines were followed in this paper^(8)^.

### Policy scenarios

#### Scenario 1

The main assumption for this scenario was that taxing foods based on their sugar content could help consumers choose foods with a lower sugar content and incentivise food manufacturers to reduce the sugar in the foods they sell^(7,9)^. In the scenario, the tax raised on a food product is assumed to depend on the quantity of sugar it contains. In addition, bigger packs and price promotions on larger packs favour higher purchase volumes and therefore consumption: people tend to purchase more products, and once the products are purchased, they are consumed^(10,11)^. Having a tax with a reduced rate for smaller packs would be expected to further reduce the quantity of sugar purchased, as this would likely incentivise smaller packs to be purchased, instead of bigger packs. Hungary implemented a tax that is proportional to the sugar content of foods in 2014. Simulations of similar taxes, applied to various sets of products, have been carried out in the UK, New Zealand and Chile^(12–14)^. Similarly, South Africa implemented a tax with a rate based on the sugar content of drinks.

Policy scenario 1 modelled the effect of a tax based on the sugar content of products on GB purchases using the 2019 Kantar Worldpanel data. The changes in total sugar and total energy purchased from these products before and after such a tax were simulated. This policy scenario was applied to sweet and chocolate confectionery, biscuits, puddings and ice creams. These categories were chosen because they have made relatively low progress towards the 20 % sugar reduction ambition of the SRP (Public Health England 2020).

Two tax rates were used: a lower one for products with a lower quantity of sugar per pack and a higher one for products above a certain threshold of sugar quantity per pack. The definition of the threshold in terms of quantity of sugar per pack allows products with a lower sugar density, and products sold in smaller packs, to sit below this threshold.

The threshold for the lower rate tax was determined using the typical portion size of the categories considered. A single threshold applied across categories was tested as this would make implementation easier. In addition, the aim of such a tax is to reduce sugar purchased by consumers, regardless of the type of product consumed. The average sugar contained in a single serve portion of products from the five categories studied varied between 17 g and 30 g (Table 1). To get a unique threshold that could work for all categories, the threshold for the lower rate was therefore set at 30 g per pack.

**Table 1.** Typical portion size and sugar density for the five categories included in policy scenario 1. Data from Kantar panel

	Typical single-serve pack size	Average sugar density	Average sugar in a single serve
Biscuits	NA*	31 g/100 g	NA
Chocolate confectionery	50 g	54 g/100 g	27 g
Ice cream	100 ml	17 g/100 ml	17 g
Puddings	100 g	19 g/100 g	19 g
Sweet confectionery	50 g	60 g/100 g	30 g

Source: Kantar Worldpanel 2019; *The typical serving size was not defined for biscuits as they are rarely sold as single pack.

In the literature, rates for similar proportional taxes on sugar content vary from about £0·10 to £0·20 per 100 g sugar content. The South African tax rate was originally set at £0·14/100 g of sugar. However, after a consultative process that resulted in concessions made to the industry, the tax was implemented at a reduced rate of about £0·11/100 g^(15)^. Prior to implementation of the SDIL in the UK, the Institute for Fiscal Policies mentioned a £0·20 tax per 100 g of sugar^(12)^. Modelling exercises in Chile used a tax rate of about £0·10/100 g of sugar in foods, while a similar exercise in New Zealand used a tax rate of £0·21/100 g^(13,14)^.

**Fig. 1.**
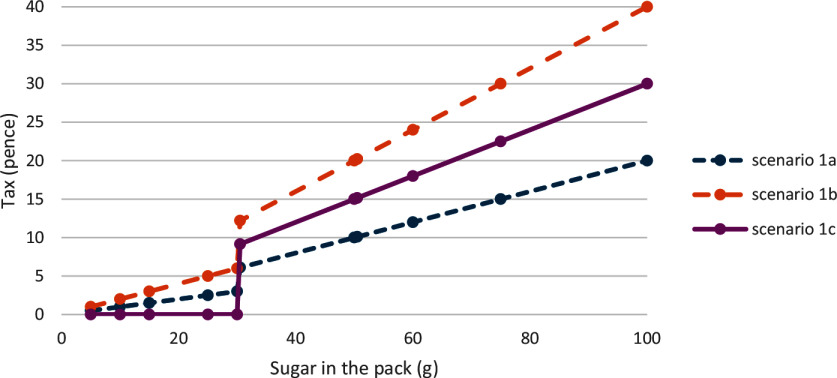
Summary of the tax rate structure applied in scenario 1.

Given the rates used in the literature, this study based the simulations on three sets of rates (Table 2). In scenario 1a, products are taxed at 0·20 p/g of sugar and at a reduced rate of 0·10 p/g if they under the 30 g per pack threshold. In scenario 1b, products under the 30 g per pack threshold are taxed at 0·20 p/g of sugar, and double this (0·40 p/g of sugar) if they are above the threshold. Scenario 1c is a flat rate tax, with a single rate of 0·30 p/g of sugar, only for products above the threshold. A product is defined as the pack that is purchased. For example, a 100 g chocolate bar with 50 g of sugar per bar will be taxed for its 50 g of sugar. The same bar sold in a 3-pack will be taxed for 150 g. Fig. 1 further shows the tax structure of scenarios 1a, 1b and 1c tested with the 30 g per pack threshold (Fig. 1).

**Table 2.** Tax rates used in scenario 1

Tax rates in pence/g of sugar	Scenario 1a	Scenario 1b	Scenario 1c
for products ≤ threshold (30 g sugar per pack)	0·10	0·20	0
for products > threshold (30 g sugar per pack)	0·20	0·40	0·30

#### Scenario 2

Using the same data source, policy scenario 2 simulates the effect of an ad valorem tax targeting ‘less healthy’ products that are identified using the UK NPM^(6)^. The NPM was developed by the FSA in 2004–2005, to provide a tool to differentiate foods and drinks appropriate for advertising to children on TV, based on their nutritional composition. The model allocates points for ‘A’ nutrients (energy, saturated fat, total sugar and Na) and ‘C’ nutrients (fruit, vegetable and nut content, fibre, and protein). The total points for ‘C’ nutrients are subtracted from the total points for ‘A’ nutrients to obtain a nutrient score. Foods scoring 4 or more points are considered ‘less healthy’ by the model. In policy scenario 2, the tax was applied to less healthy products (scoring 4 or more points) in food categories that were included in the SRP.

The NPM score was calculated for all products included in the SRP food categories. Kantar Worldpanel does not have information on fruit/vegetable/nut (FVN) content, and therefore the FVN content points were not included in calculating the overall score. Excluding this component is unlikely to change healthier/less healthy food category of foods included in this study as, to score any points, the FVN content of the food has to be higher than 40 %. Considering all the food categories included in scenario 2 are low in FVN content, assuming these food score 0 in the FVN dimension is reasonable. The products in each food category were then divided into two subcategories: those that were classified as less healthy (score ≥ 4) and those that were categorised as healthier (score < 4)^(6)^. The SRP has ten food categories (i.e. biscuits, breakfast cereal, cakes, chocolate confectionery, ice cream, morning goods, puddings, sweet spreads and sauces, sweet confectionery, and yogurts), so twenty subcategories in total were included in scenario 2.

Table 3 presents the numbers of products in each food category. There are more than 72 % healthier products in the yogurt category, more than 20 % in the morning goods category, more than 10 % in the ice cream and pudding categories, between 1 and 2 % in the biscuit, cakes, sweet spread and sauce, and sweet confectionery categories, and less than 1 % in the chocolate confectionery categories.

**Table 3.** Numbers (*n*) of less healthy and healthier products in the Kantar Worldpanel 2019

SRP category	*n* of healthier products	*n* of less healthy products	% healthier products
Biscuits	58	3391	2
Breakfast cereals	1056	500	68
Cakes	20	1049	2
Chocolate confectionery	8	4116	0
Ice cream	174	1287	12
Morning goods	145	372	28
Puddings	373	2311	14
Sweet spreads and sauces	4	391	1
Sweet confectionery	41	3036	1
Yogurts	1102	420	72

SRP, Sugar Reduction Programme.

Data source: 2019 Kantar Worldpanel, products that are missing nutrient information were excluded from the calculation.

In terms of tax rate, a 20 % increase in the price of less healthy foods was used in the main analysis of scenario 2, as evidence suggests that a 20 % tax can increase the price sufficient to produce purchases of taxed products^(9,16)^. Sensitivity analyses was conducted using a lower tax rate of 10 % and a higher tax rate of 30 %, respectively.

### Price elasticity

The change in the quantity of products purchased following an increase in their price as a result of a tax was simulated using price elasticities. As estimating price elasticities require detailed household data, which we did not have access to, estimates of the category price elasticities were taken from the study by Cornelsen and colleagues^(7)^. Cornelsen *et al.* estimated category-level price elasticities using a demand model based on UK purchases data from Kantar Worldpanel covering the period from January 2012 to December 2013^(7)^. A major distinction between the two studies is that this study focuses on categories covered in the SRP, while Cornelsen *et al.* included all food categories available in the Kantar data. Specifically relevant to scenario 2, in Cornelsen *et al.* ^(7)^, some food categories, namely bread and morning goods, cereal and cereal bars, and dairy products, were divided into healthier and less healthy subcategories, and price elasticities were estimated with respect to each subcategory. However, for sweet snacks, desserts and puddings, the differentiation between less healthy and healthier products was not made because most products (> 80 %) in these categories were less healthy. Where Cornelsen *et al.* ^(7)^ has made a differentiation, the price elasticity of less healthy products in that food category was used in this study. Otherwise, the price elasticity of the whole category was used.

Price elasticities at product level had to be estimated for this study, knowing that price elasticity at the level of individual products is higher than the price elasticity for a category as consumers are more likely to switch between products within a category than between categories. Product-level price elasticities were defined according to each product’s standardised value (*z*-score); products that had a higher increase in price than the average change in price for the category received a negative price elasticity, while products that had a change in price that was smaller than the average for the category received a positive price elasticity. That way, the sum of the changes in demand for each product in a category equals the change in demand for the category.

Table 4 presents the price elasticity of each less healthy food subcategory. In general, the value of own-price elasticity for food categories in the SRP is about −0·8, indicating an inelastic demand for these food products, that is, changes in the price do not result in much change in the quantity demanded. The own-price elasticity of demand is the percentage change in the demand of a good or service divided by the percentage change in the price. This calculates how responsive demand is to a change in price.

**Table 4.** Price elasticity of less healthy food subcategory (95 % CI)

Food subcategory	Own-price elasticity	95 % CI (lower), 95 % CI (upper)
Biscuits (less healthy)	–0·860	–0·896, −0·824
Breakfast cereal (less healthy)	–0·896	–0·950, −0·842
Cakes (less healthy)	–0·824	–0·858, −0·790
Chocolate confectionery (less healthy)	–0·860	–0·896, −0·824
Ice cream (less healthy)	–0·813	–0·846, −0·781
Morning goods (less healthy)	–0·824	–0·858, −0·790
Puddings (less healthy)	–0·813	–0·846, −0·781
Spreads and sauces (less healthy)	–0·813	–0·846, −0·781
Sweet confectionery (less healthy)	–0·860	–0·896, −0·824
Yogurts (less healthy)	–0·783	–0·824, −0·741

Data source: Cornelsen *et al.* (2019).

### Statistical analysis

A 100 % pass-through rate was assumed in both scenarios 1 and 2, indicating that the price of products increased by exactly the amount of the tax. The changes in price and total spending on food categories as a result of each proposed tax, weighted by the sales of each product within the given food category, were calculated. The changes in total sugar and energy purchases were simulated using price elasticities. Based on the price elasticity, the change in purchases of products, and in purchases of total sugar and total energy, following taxes proposed in scenarios 1 and 2, were computed for each scenario. In the calculation, it was assumed that the purchases of products in other food categories or untaxed products would not be affected by the tax. A sensitivity analysis was conducted for both policy scenarios using the 95 % CI of the own-price elasticity estimates provided in the Cornelsen *et al.* (2019) study^(7)^ to assess the upper/lower boundaries of point estimates produced in the present study.

Reformulation by businesses, as a response before the implementation of proposed tax, was taken into consideration in both scenarios as reformulation is particularly relevant in this context. The amount of revenue raised by a tax can be modified by reformulation of affected products. In scenario 1, the tax is proportional to the amount of total sugar in a pack. This tax structure is expected to promote reformulation, as a reduction in the sugar content of a product would be translated to a reduction in the tax rate applicable and the amount of revenue raised. Similarly, in scenario 2, the tax may encourage manufacturers to improve the overall nutrient quality of their less healthy products to achieve a NPM score that is below the taxation threshold (i.e. becoming healthier products).

In scenario 1, the effect of reformulation in response to taxation was only tested in scenario 1c (unique tax rate). This was because, compared with scenarios 1a and 1b, scenario 1c provides the largest incentive for reformulation due to drinks with sugar content below the 30 g pack threshold are not taxed. Reformulation was modelled as a reduction in the sales-weighted sugar content of the categories evaluated (column 2 of Table 5). The reductions were designed based on what was observed in the voluntary SRP and used insights about the technical feasibility of reformulation of the categories used. The model assumed that all manufacturers would reformulate by the same percentage across the whole category (column 4 of Table 5). The estimated percentage reduction in sales-weighted average sugar content had 2015 as a baseline. The reductions from 2019 (year for which the scenarios were simulated) were calculated considering the reformulation that could have occurred between 2015 and 2019.

**Table 5.** Modelled reformulation percentage for products in taxed categories

	SWA^*^ total sugar in 2015 (g/100 g)	% Reformulation objective for the category	SWA total sugar level 2019	% Reformulation from 2019 for each product	SWA total sugar after the tax (g/100 g)
Biscuits	31·6	10 %	31·0	8·2 %	28·4
Chocolate confectionery	54·4	5 %	53·9	4·2 %	51·7
Ice creams	18·6	15 %	17·46	9·5 %	15·8
Puddings	18·3	5 %	18·7	7·1 %	17·4
Sweet confectionery	61·2	5 %	60·4	3·8 %	58·1

* SWA: sales-weighted average.

In scenario 2, to take into account reformulation, we have assessed whether a reduction in either saturated fat, salt or sugar of the same magnitude as the reduction envisaged in scenario 1 would change the classification of a less healthy food product from less healthy to healthier (lowering the UK-NPM score below 4). The products whose reformulation had the potential to exclude them from the tax base were assumed to be reformulated and therefore not taxed. The analysis assumed that: first, reformulation would take place for sugar, saturated fat and Na individually, but not all at the same time; second, reformulation of sugar and saturated fat would affect the energy content of the products being reformulated at a conversion rate of 16·7 kJ/g for sugar and 37·7 kJ/g for saturated fat; lastly, the magnitude of saturated fat and Na reformulation was assumed to be the same as that for sugar. However, reformulation was only considered as an additional sensitivity analysis to the main analysis in scenario 2, because this analysis was based on strong assumptions that may deviate from the real world.

## Results

### Scenario 1

Taxing products at a rate of 0·1 p/g of sugar for smaller packs and 0·2 p/g of sugar for larger packs corresponded to an average price per pack increase of 12–31 % across categories (Table 6). Taxing at higher rates (0·2 p/g of sugar and 0·4 p/g of sugar in bigger pack) corresponded to increases in price per pack of, on average, 20 % to 60 %. Taxing with a flat rate of 0·3 p/g of sugar, exempting products below the threshold, created average price increases of between 19 % and 46 % (with no reformulation). Biscuits (49 %) and sweet confectionery (52 %) had the largest price increases. This result is explained by the fact that these two categories had, at baseline, a lower price per gram of sugar per 100 g of product. Adding reformulation to scenario 1c resulted in a smaller increase in price (assuming a 100 % pass-through). This result is expected with reformulation, as manufacturers are expected to reformulate their products to mitigate the price increase of their products. The effect was stronger for biscuits where a 10 % reduction in sales-weighted sugar content was modelled. Spending increased between 6 % and 22 %, depending on the scenarios. Reformulation had almost no impact on the change in spending, despite changes in prices. Change in energy purchased was not reported for scenario 1c with and without reformulation, because we only modelled reformulation of sugar, not other nutrients, and what would have happened to energy in scenario 1c was therefore unknown. We assumed that reformulation of sugar would not lead to any changes in energy purchased.

**Table 6.** Results of the simulated tax on percentage change in sales-weighted mean price, volume and sugar purchased. Data from Kantar panel

Tax design	Category	Change in price/pack	Change in price/100 g	Change in spending	Change in volume purchased	Change in sugar purchased	Change in energy purchased
0·1 p/g if sugar < 30 g, 0·2 p/g if sugar > 30 g	Biscuits	31 %	34 %	13 %	–15 %	–15 %	–15 %
	Chocolate confectionery	20 %	24 %	10 %	–12 %	–13 %	–11 %
	Ice cream	16 %	28 %	13 %	–12 %	–13 %	–11 %
	Puddings	14 %	16 %	6 %	–9 %	–15 %	–14 %
	Sweet confectionery	30 %	37 %	12 %	–18 %	–23 %	–18 %
0·2 p/g if sugar < 30 g, 0·4 p/g if sugar > 30 g	Biscuits	61 %	67 %	22 %	–27 %	–26 %	–27 %
	Chocolate confectionery	38 %	47 %	17 %	–21 %	–23 %	–19 %
	Ice cream	25 %	53 %	21 %	–21 %	–23 %	–21 %
	Puddings	24 %	31 %	9 %	–17 %	–28 %	–26 %
	Sweet confectionery	53 %	71 %	18 %	–31 %	–38 %	–30 %
0·3 p/g if sugar > 30 g	Biscuits	46 %	49 %	17 %	–21 %	–20 %	–22 %
	Chocolate confectionery	29 %	34 %	12 %	–16 %	–18 %	–15 %
	Ice cream	21 %	41 %	17 %	–17 %	–19 %	–16 %
	Puddings	19 %	22 %	6 %	–12 %	–20 %	–21 %
	Sweet confectionery	42 %	52 %	14 %	–25 %	–31 %	–25 %
0·3 p/g if sugar > 30 g, with reformulation	Biscuits	42 %	45 %	16 %	–20 %	–26 %	NA
	Chocolate confectionery	28 %	33 %	12 %	–16 %	–21 %	NA
	Ice cream	20 %	37 %	16 %	–16 %	–25 %	NA
	Puddings	18 %	20 %	6 %	–12 %	–25 %	NA
	Sweet confectionery	41 %	50 %	14 %	–24 %	–32 %	NA

All changes are sales-weighted averages; NA, not applicable.

**Fig. 2.**
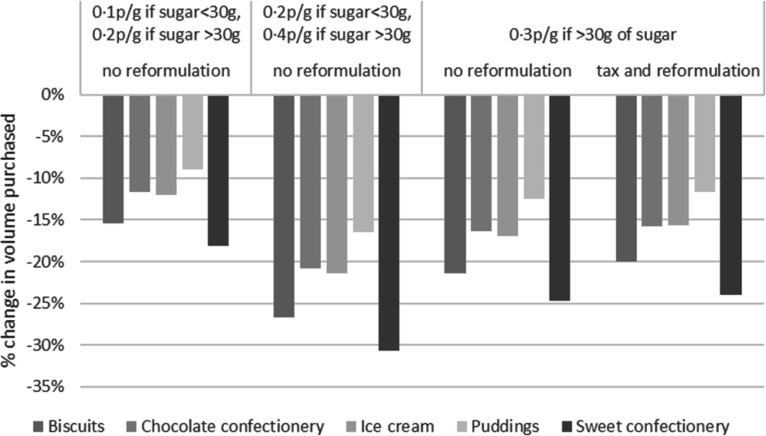
Change in total volume purchased.

The simulated change in volume purchased after the tax ranged from −8 % to −27 % (Table 6 and Fig. 2). Categories with the highest decrease across all scenarios were sweet confectionery and biscuits. This was linked to a greater change in price for these categories that have the same price elasticity (Table 6). Reformulation led to smaller decreases in volume purchased for all categories (compared with the same tax structure with no reformulation (Fig. 2)). This is explained by the fact that if reformulation reduces sugar content, it reduces the tax for products (the incentive for reformulation); therefore, there is a smaller increase in price resulting in a smaller change in quantities purchased.

**Fig. 3.**
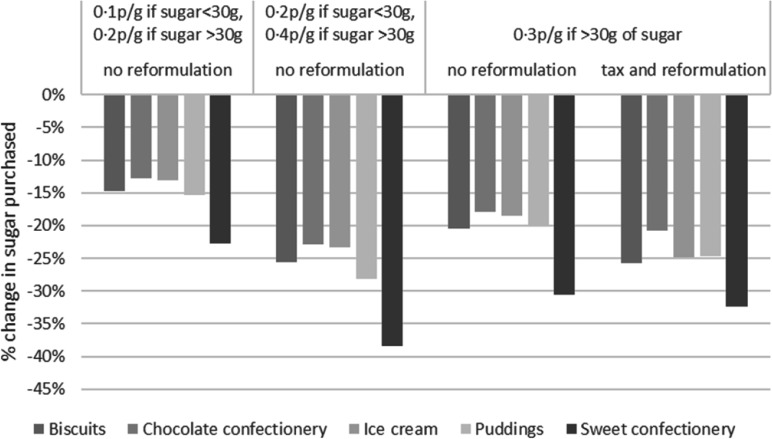
Change in sugar quantities purchased.

The quantity of sugar purchased decreased by between 13 % and 38 % (Table 6 and Fig. 3). The largest decreases were for the sweet confectionery category (–23 % for tax rate at 0·10 then 0·20 p/g, –38 % for tax rate at 0·2 then 0·40 p/g, 31 % for the flat tax rate at 0·3 p/g without reformulation and 32 % for the flat tax rate at 0·3 p/g with reformulation). Decreases for the three other categories were similar. The reformulation of products led to further decreases in sugar purchased for all categories as reformulation reduced the quantity of sugar in the product. The sensitivity analysis performed for policy scenario 1a, using the lower/upper bounds of own-price elasticities, showed that changes in the sales-average volume, sugar quantity of energy were robust for scenario 1a (Table 7). Given that all scenarios (i.e. 1a, 1b and 1c) used the same price elasticity, it is assumed that all policy scenarios produced robust estimates.

**Table 7.** Sensitivity analysis for policy scenario 1a

Tax design	Category	Change in price/pack		Change in price/100		Change in spending		Change in volume		Change in sugar		Change in energy	
0·10 p/g if sugar < 30 g, 0·20 p/g if sugar > 30 g	Biscuits	31 %	30 %; 32 %	34 %	33 %; 35 %	13 %	13 %; 13 %	–15 %	–15 %; −16 %	–15 %	–14 %; −15 %	–15 %	–15 %; −16 %
	Chocolate confectionery	20 %	20 %; 20 %	24 %	25 %; 24 %	10 %	10 %; 10 %	–11 %	–12 %; −12 %	–12 %	–13 %; −13 %	–10 %	–11 %; −11 %
	Ice cream	16 %	16 %; 16 %	28 %	28 %; 29 %	13 %	13 %; 13 %	–12 %	–12 %; −12 %	–13 %	–13 %; −14 %	–11 %	–11 %; −12 %
	Puddings	14 %	14 %; 14 %	16 %	16 %; 16 %	6 %	6 %; 5 %	–9 %	–9 %; −9 %	–15 %	–15 %; −16 %	–14 %	–14 %; −15 %
	Sweet confectionery	30 %	30 %; 30 %	37 %	36 %; 37 %	12 %	12 %; 12 %	–18 %	–17 %; −19 %	–23 %	–22 %; −23 %	–18 %	–17 %; −19 %

Data source: Kantar Worldpanel 2019; Note: all changes are sales-weighted averages.

### Scenario 2

Table 8 shows the baseline average price for each subcategory, and the corresponding after-tax price for each less healthy subcategory, following a 20 % ad valorem tax on less healthy products. It is worth mentioning that, at baseline, the sales-weighted average price of healthier products was already lower than that of less healthy products within some food categories. This applied to breakfast cereals, cakes, ice cream, morning goods, puddings, sweet spreads and sauces, and yogurts. Post-tax the average price of less healthy products was higher than that of healthier products in almost all food categories, except for biscuits and sweet confectionery which already had a much higher baseline average price for the healthier products.

**Table 8.** Baseline and post-tax price levels of each food subcategory

	Baseline average price (£/kg)		Post-tax average price (£/kg)	
Food category	Healthier	Less Healthy	Healthier	Less healthy
Biscuits	£8·52	£4·16	£8·52	£5·00
Breakfast cereals	£2·99	£3·35	£2·99	£4·02
Cakes	£4·34	£5·28	£4·34	£6·34
Chocolate confectionery	£8·94	£8·60	£8·94	£10·32
Ice cream	£1·98	£3·04	£1·98	£3·65
Morning goods	£2·54	£3·64	£2·54	£4·36
Puddings	£2·22	£4·48	£2·22	£5·38
Spreads and sauces	£1·56	£4·67	£1·56	£5·60
Sweet confectionery	£12·40	£5·55	£12·40	£6·66
Yogurts	£2·57	£2·84	£2·57	£3·41

Sales weighted average prices used.

Data Source: 2019 Kantar Worldpanel; Note: Sales-weighted average prices used.

Total spending (after-tax price multiplied by quantity purchased) on less healthy products increased by 1·8 % to 3·8 % (Table 9), depending on the food category. Total spending still increased because of the inelastic demand for products being studied. As the demand for less healthy products decreased at a slower rate than the increase in price, total spending increased.

**Table 9.** Post-tax changes in total expenditure on less healthy products in comparison with the baseline

Product category	Total spending on less healthy products (£1000)		Change in total spending on less healthy products (%)
	Baseline	Post-tax	Post-tax in comparison with baseline
Biscuits	2 223 631	2 276 755	2·4 %
Breakfast cereal	536 362	545 823	1·8 %
Cakes	849 438	875 108	3·0 %
Chocolate confectionery	3 143 066	3 218 156	2·4 %
Ice cream	973 915	1 005 245	3·2 %
Morning goods	527 621	543 565	3·0 %
Puddings	1 161 640	1 199 009	3·2 %
Spreads and sauces	204 836	211 426	3·2 %
Sweet confectionery	895 904	917 308	2·4 %
Yogurts	348 262	361 330	3·8 %

Data source: 2019 Kantar Worldpanel.

Due to decreased demand for less healthy products after the application of a tax, total sugar, total energy, total saturated fat and total Na purchased showed substantial reductions (Tables 10–12). Relative to the baseline, total sugar, total energy, total saturated fat and total Na purchased from less healthy products decreased by between 13·5 % and 15·2 % (Tables 10–12)). This was due to all calculations were based on the same set of price elasticity. Keeping the purchase of healthier products constant, at the category level, a 20 % tax on less healthy products would reduce total sugar purchased by 4·3 % to 14·7 % (Table 10), total energy purchased by 4·7 % to 14·7 % (Table 11), total saturated fat purchased by 7·2 % to 14·7 % and total Na purchased by 3·2 % to 14·7 %. Results from the sensitivity analysis using the upper/lower limits of own-price elasticities were consistent with the main results (Table 12). Results of 10 % and 30 % tax rates are presented Appendix Tables E2–E4. Overall, taxing less healthy products was estimated to have stronger effects on food categories that have a higher proportion of less healthy products, such as chocolate and sweet confectionery.

**Table 10.** Change in total sugar, energy, saturated fat and sodium purchased without considering formulation

	Sugar		Energy		Saturated fat		Na	
Product category	Total sugar purchased before tax (1000 kg)	Change in sugar purchased (%)	Total energy purchased before tax (10^9^ kJ)	Change in energy purchased (%)	Total saturated fat purchased before tax (1000 kg)	Change in saturated fat purchased (%)	Total Na purchased before tax (1000 kg)	Change in saturated fat purchased (%)
Biscuits	167 316	–14·6 %	10 672	–14·5 %	54 397	–14·6 %	1297	–14·6 %
Breakfast cereal	71 652	–8·6 %	8032	–5·2 %	6625	–8·8 %	815	–6·2 %
Cakes	54 718	–13·6 %	2870	–13·5 %	12 452	–14·1 %	304	–13·1 %
Chocolate confectionery	197 303	–14·7 %	7810	–14·7 %	57 186	–14·7 %	409	–14·7 %
Ice cream	65 193	–12·9 %	2727	–13·2 %	22 694	–13·5 %	183	–12·5 %
Morning goods	34 001	–8·7 %	3169	–8·3 %	7589	–12·6 %	958	–8·5 %
Puddings	72 155	–11·5 %	3398	–12·1 %	18 052	–12·9 %	310	–11·0 %
Spreads and sauces	12 705	–14·0 %	1014	–14·0 %	3534	–14·0 %	77	–14·0 %
Sweet confectionery	98 257	–14·7 %	2583	–14·6 %	2933	–14·6 %	146	–14·6 %
Yogurts	56 949	–4·3 %	2037	–4·7 %	8892	–7·2 %	332	–3·2 %

Assuming no change in purchases of healthy products in the same category.

Data Source: 2019 Kantar Worldpanel.

**Table 11.** Change in total sugar, energy, saturated fat and sodium purchased considering formulation

	Sugar		Energy		Saturated fat		Na	
Product category	Total sugar purchased before tax (1000 kg)	Change in sugar purchased (%)	Total energy purchased before tax (10^9^ kJ)	Change in energy purchased (%)	Total saturated fat purchased before tax (1000 kg)	Change in saturated fat purchased (%)	Total Na purchased before tax (1000 kg)	Change in saturated fat purchased (%)
Biscuits	167 316	–14·5 %	10 672	–14·5 %	54 397	–14·6 %	1297	–14·5 %
Breakfast cereal	71 652	–7·9 %	8032	–4·7 %	6625	–8·4 %	815	–5·5 %
Cakes	54 718	–13·6 %	2870	–13·5 %	12 452	–14·1 %	304	–13·1 %
Chocolate confectionery	197 303	–14·7 %	7810	–14·7 %	57 186	–14·7 %	409	–14·7 %
Ice cream	65 193	–12·5 %	2727	–12·9 %	22 694	–13·3 %	183	–12·2 %
Morning goods	34 001	–8·0 %	3169	–6·6 %	7589	–12·3 %	958	–4·6 %
Puddings	72 155	–11·3 %	3398	–12·0 %	18 052	–12·8 %	310	–10·9 %
Spreads and sauces	12 705	–14·0 %	1014	–14·0 %	3534	–14·0 %	77	–14·0 %
Sweet confectionery	98 257	–14·7 %	2583	–14·6 %	2933	–14·6 %	146	–14·6 %
Yogurts	56 949	–3·7 %	2037	–4·1 %	8892	–6·5 %	332	–2·7 %

**Table 12.** Sensitivity analysis – lower and upper limits of post-tax changes at the food category level

Post-tax estimates	Change in total sugar purchased		Change in total energy purchased		Change in total saturated fat purchased		Change in total Na purchased	
	%		%		%		%	
	Lower	Upper	Lower	Upper	Lower	Upper	Lower	Upper
Biscuits	–15·1 %	–14·0 %	–15·1 %	–14·0 %	–15·2 %	–14·1 %	–15·1 %	–14·1 %
Breakfast cereal	–9·0 %	–8·1 %	–5·4 %	–4·9 %	–9·2 %	–8·3 %	–6·5 %	–5·9 %
Cakes	–14·1 %	–13·1 %	–13·9 %	–13·0 %	–14·6 %	–13·6 %	–13·6 %	–12·6 %
Chocolate confectionery	–15·2 %	–14·1 %	–15·2 %	–14·1 %	–15·2 %	–14·1 %	–15·2 %	–14·1 %
Ice cream	–13·4 %	–12·5 %	–13·6 %	–12·7 %	–14·0 %	–13·0 %	–13·0 %	–12·1 %
Morning goods	–9·0 %	–8·4 %	–8·5 %	–8·0 %	–13·1 %	–12·2 %	–8·8 %	–8·2 %
Puddings	–11·9 %	–11·1 %	–12·5 %	–11·6 %	–13·4 %	–12·5 %	–11·4 %	–10·7 %
Spreads and sauces	–14·5 %	–13·5 %	–14·5 %	–13·5 %	–14·5 %	–13·5 %	–14·5 %	–13·5 %
Sweet confectionery	–15·2 %	–14·1 %	–15·1 %	–14·0 %	–15·1 %	–14·1 %	–15·1 %	–14·1 %
Yogurts	–4·5 %	–4·1 %	–4·9 %	–4·5 %	–7·5 %	–6·8 %	–3·3 %	–3·0 %

Assuming no change in purchases of healthy products in the same category; reformulation was not considered in the sensitivity analysis.

Data source: 2019 Kantar Worldpanel.

## Discussion

In this study, two different tax scenarios were modelled: one based on the quantity of sugar in packs and the other on the nutrient profile of food products. For both scenarios, the analysis was completed on categories, or a subset of categories included in the UK voluntary SRP. In scenario 1, both a flat and a two-level tax based on sugar content per pack would result in a reduced quantity of sugar being purchased from taxed categories. Data from this analysis show that using the same sugar per pack threshold across product categories in the two-level tax structure is feasible. The value of the tax threshold has a small influence on the total volume of products and sugar quantity purchased. The modelling of the reformulation of products under the flat tax structure showed that reformulation would not drive further change in spending, and the purchasing of higher quantities of products but lower sugar quantities, compared with the same flat tax rate scenario with no reformulation. In policy scenario 2, taxation based on the UK NPM at a rate of 20 % reduced total sugar and total energy purchased from less healthy products by 13·5–15·2 %. It reduced total sugar and energy purchased from all categories included by 4·3–14·7 % and 4·7–14·7 %, respectively. Both scenarios demonstrated the potential of using fiscal policies to reduce household purchases of sugar and energy.

Comparing the two scenarios, the effect of taxing sugar content at 0·10 p/g in small packs and 0·20 p/g in bigger packs was similar (i.e. the difference was less than 5 %) to that of taxing unhealthy products at a rate of 20 % for biscuits (–15·0 % *v*. −14·6 % in sugar purchased and −15·0 % *v*. −14·5 % in energy purchased), chocolate confectionery (–13·0 % *v*. −14·7 % in sugar purchased and −11·0 % *v*. −14·7 % in energy purchased), ice cream (–13·0 % *v*. −12·9 % in sugar purchased and −11·0 % *v*. −13·2 % in energy purchased) and pudding (–15·0 % *v*. −11·5 % in sugar purchased and −14·0 % *v*. −12·1 % in energy purchased). However, the same does not hold for sweet confectionery (–23·0 % *v*. −14·7 % in sugar purchased and −18 % *v*. −14·6 % in energy purchased). This is because the price of products in the sweet confectionery category (in which sugar content is high) would have been affected by a sugar content tax most, compared with products in other food categories. The effect of taxing sugar content at 0·20 p/g then 0·40 p/g on reducing sugar and energy purchased is stronger than that of a 20 % ad valorem tax on less healthy products for all common food categories in scenarios 1 and 2. This is because the impact of tax on food price is higher in scenario 1 (when 0·20 p/g and 0·40 p/g tax rates are used) than in scenario 2.

To the best of our knowledge, we add to the existing body of literature and academic and political debates by simulating two innovative and feasible fiscal policies that aim at reducing sugar and energy consumption and providing insights into the potential impact of such policies. This study explores different policy options, including a set of narrow-based exercise tax on sugar content within food products, as well as a broader-based ad valorem tax on less healthy food products based on the UK NPM score which takes into account the overall nutritional composition of food products. It provides evidence that fiscal policies have the potential to reduce consumption of less healthy food products.

The study has several limitations: first, a 100 % pass-through of tax was assumed. It is anticipated that the effect size would decrease if the pass-through rate was lower. Second, in scenario 1, between-category substitutions were not modelled, and in scenario 2, it was assumed that purchases of healthier products remained constant following increases in price of less healthy products, which is not likely to be the case in real-world setting. Modelling changes in the purchase of healthier products is complex and requires further dedicated research. It demands a deep understanding of consumer behaviour when facing increases in the price of less healthy products. When the price of less healthy products increases, a consumer may (1) switch to a cheaper less healthy alternative in the same food category, (2) switch to a healthier product in the same food category, (3) switch to a product in another food category or (4) continue buying the product. Depending on the products consumers purchase as substitutes, the overall impact on energy content/sugar purchased may be limited, especially if consumers switch to products with a similar level of energy content/sugar. Additionally, there are other factors beyond price, including but not limited to taste preferences, brand loyalty and awareness of health implications that could influence consumer’s behaviour. It is possible that consumers may not significantly change their purchases due to these factors despite the tax. However, taxation of less healthy products is likely to still encourage consumers to buy healthier options as simulated in this study. Third, only food categories included in the SRP were analysed in these policy scenarios. Although the NPM has been widely used in nutrition-related policies, it was not originally developed for use in taxation policies. A tax based on the NPM could be applied in other food categories to provide consumers with incentives to consume healthier products. Fourth, in these policy scenarios, purchase data rather than consumption data were used, and, due to food waste, the estimated impact of tax on energy content/sugar is likely to be upwardly biased. Finally, the calculation used to derive the price elasticity of individual products from the price elasticity of the category was rather basic. This was because of limited information on individual product price elasticities or consumer demand for individual products. As such, details at the individual product level are inaccurate and thus beyond the scope of this study.

While the implementation of SDIL has been successful in reducing the contributions drinks make to sugar consumption in the UK, the effectiveness of the levy might be further increased by improving its design, for instance, by extending the tax base to include additional product categories. Further public health benefits may also derive from the use of taxes based on the overall nutrient profile of foods, applied to all or a wide range of foods, such as the one simulated in policy scenario 2. As shown in the results section, both taxation scenarios reduced sugar and energy purchased. On the other hand, the taxes simulated in this study would require a comprehensive system for recording and regularly updating nutrient information for all foods to enable calculation of the NPM score and monitoring of compliance to the tax. It is also important to highlight that both tax scenarios have been found to increase consumer spending, which may lead to regressive impacts, depending on food purchase patterns in different socio-economic groups. The distributional impacts of a tax can be mitigated in some cases, or even reversed, with an appropriate tax design. Therefore, the implementation of tax policies modelled on the scenarios explored in this study would require additional analyses of consumer behaviour in different socio-economic groups and simulations of how alternative tax designs would impact on such different groups.
